# Well-Differentiated Squamous Cell Carcinoma Arising in Chronic Hypertrophic Lichen Planus: A Case Report

**DOI:** 10.7759/cureus.110736

**Published:** 2026-06-12

**Authors:** Lathika Premkumar, Sai Preethi P, Murugan Sundaram, Adikrishnan Swaminathan

**Affiliations:** 1 Dermatology, Venereology and Leprosy, Sri Ramachandra Institute of Higher Education and Research, Chennai, IND

**Keywords:** cutaneous neoplasm, dermatopathology, hypertrophic lichen planus, malignant transformation, p53, pseudoepitheliomatous hyperplasia, squamous cell carcinoma

## Abstract

Squamous cell carcinoma (SCC) arising in chronic hypertrophic lichen planus represents a rare but clinically relevant malignant transformation, presenting diagnostic complexities attributed to morphological similarities with other dermatoses. The paucity of documented cases necessitates comprehensive documentation of clinical and histopathological findings. A 56-year-old woman presented with a six-year history of a progressively enlarging, pruritic, hyperpigmented plaque localized to the right lower limb. Clinical examination revealed a well-defined, indurated plaque measuring 5×3 centimeters with areas of central erosion over the lateral aspect of the right calf. Histopathological analysis of a punch biopsy specimen demonstrated well-differentiated SCC characterized by epidermal cells in nests showing keratinization and formation of keratin pearls, with evidence of dermal invasion. The differential diagnostic considerations included hypertrophic lichen planus, chromoblastomycosis, and SCC. This case emphasizes the need for vigilance in chronic hypertrophic lichen planus to detect malignant transformation.

## Introduction

Hypertrophic lichen planus (HLP) represents a distinct clinical variant of lichen planus, characterized by hyperkeratotic papules and plaques with predilection for the lower extremities [1]. The condition demonstrates an estimated prevalence of 0.5-2% within the general population, with the hypertrophic variant comprising 4.5-19% of all lichen planus cases [2].

The pathogenic mechanisms underlying HLP involve T-cell-mediated autoimmune responses directed against basal keratinocytes. This sustained inflammatory milieu, coupled with repetitive trauma and chronic irritation, establishes a microenvironment conducive to neoplastic transformation. Molecular analyses have also identified p53 tumor suppressor gene mutations [3].

Cutaneous squamous cell carcinoma (SCC) arising from HLP remains relatively rare, with reported incidence rates ranging from 0.4% to 1.2% [4]. The diagnostic complexities in differentiating early carcinomatous changes from the underlying inflammatory process necessitate a high index of clinical suspicion and prompt histopathological evaluation.

This report documents an instance of well-differentiated SCC developing within chronic HLP, emphasizing the critical importance of histopathological assessment in chronic dermatoses exhibiting atypical features.

## Case presentation

A 56-year-old post-menopausal female patient presented with a six-year history of a progressively enlarging, hyperpigmented, pruritic lesion localized to the right lower extremity. Symptoms of malignant transformation, like pain, ulceration, bleeding, and fixity to underlying structures, were ruled out. Past medical history was significant for systemic hypertension, managed with amlodipine 5 mg daily. The patient denied any history of antecedent trauma, lesional discharge, or constitutional symptoms. Therapeutic history included a one-year course of topical corticosteroids, with intermittent application of salicylic acid and topical mupirocin preparations.

**Figure 1:**
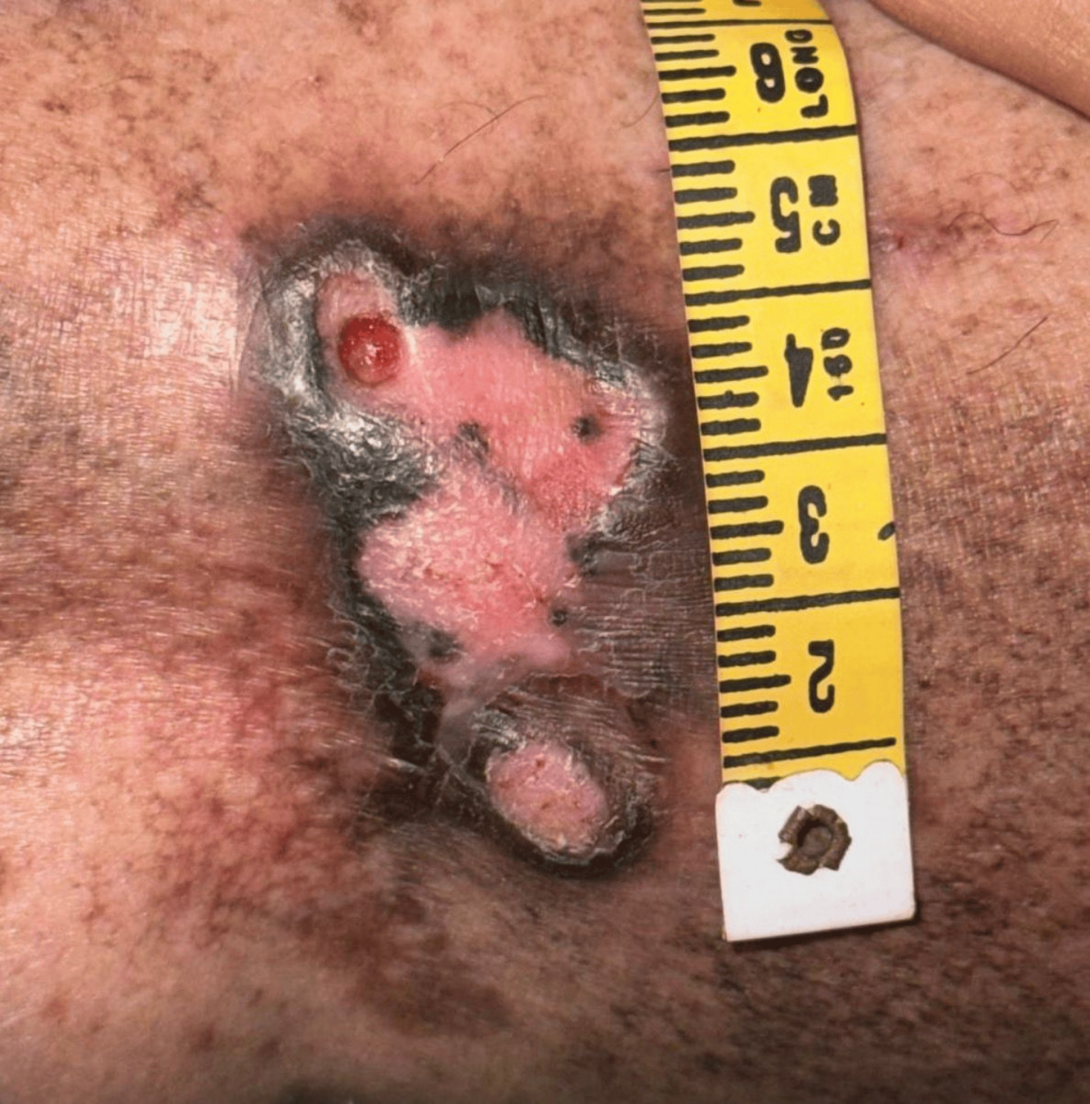
Well-demarcated, hyperpigmented, indurated plaque measuring 3.5×3 cm with areas of central erosion over the lateral aspect of the right calf

Vital parameters and general and systemic examinations were unremarkable. Regional lymphadenopathy was ruled out. Dermatological examination revealed a solitary, well-demarcated, hyperpigmented, indurated plaque measuring 3.5 × 3 cm with irregularities in the margin and areas of central erosion over the lateral aspect of the right calf (Figure 1).

Examination of the oral mucosa, nails, and scalp showed no significant abnormalities. 

**Figure 2:**
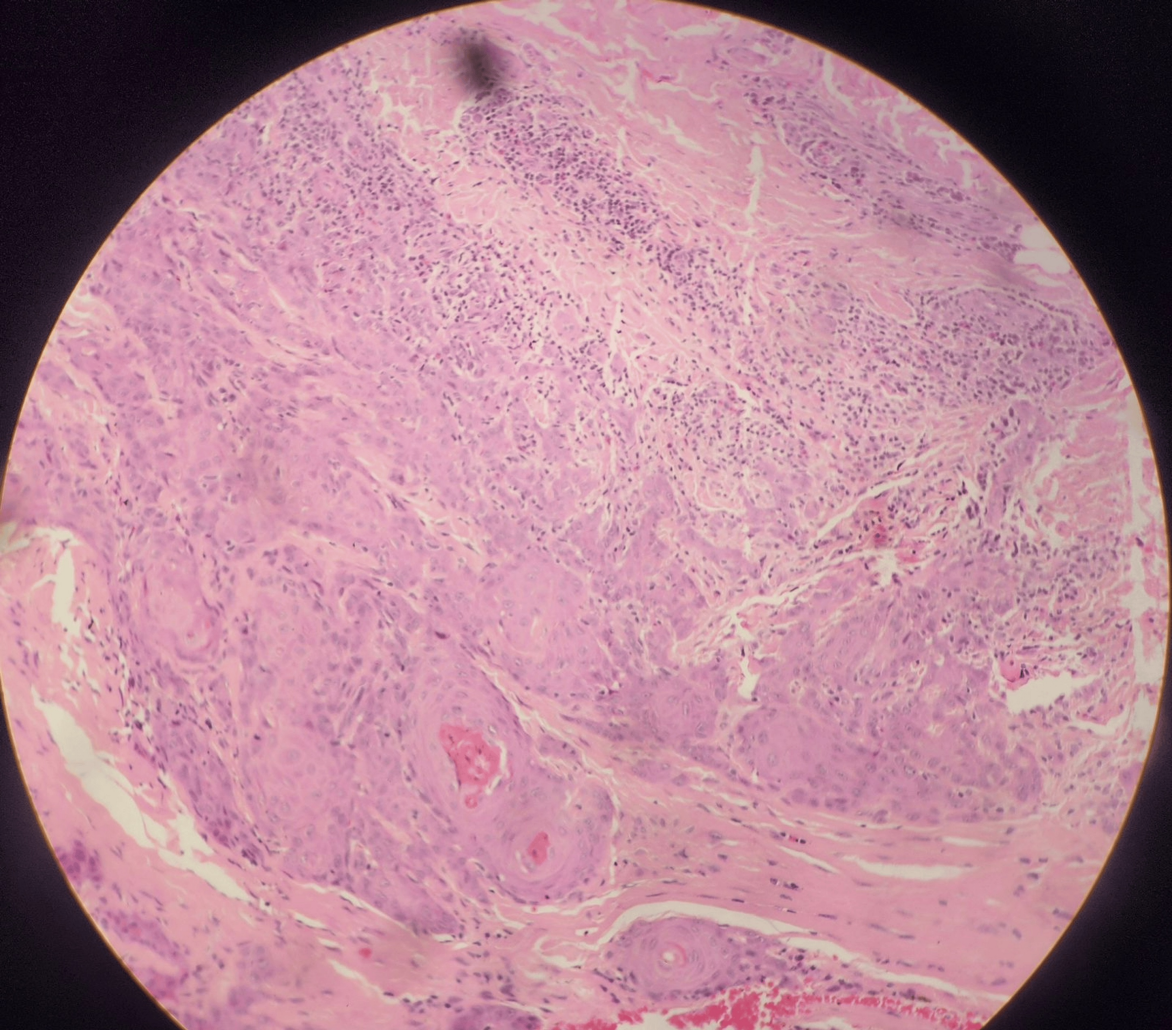
Low-power examination reveals an infiltrative neoplasm within the dermis composed of irregular nests and islands of atypical squamous epithelial cells

Laboratory investigations were performed as per institutional protocols. A 3.5 mm skin punch biopsy was obtained from the plaque, post-procedure being uneventful. Figure 2 shows the low-power examination of the dermis, showing infiltrating nests and islands of atypical squamous epithelial cells.

**Figure 3:**
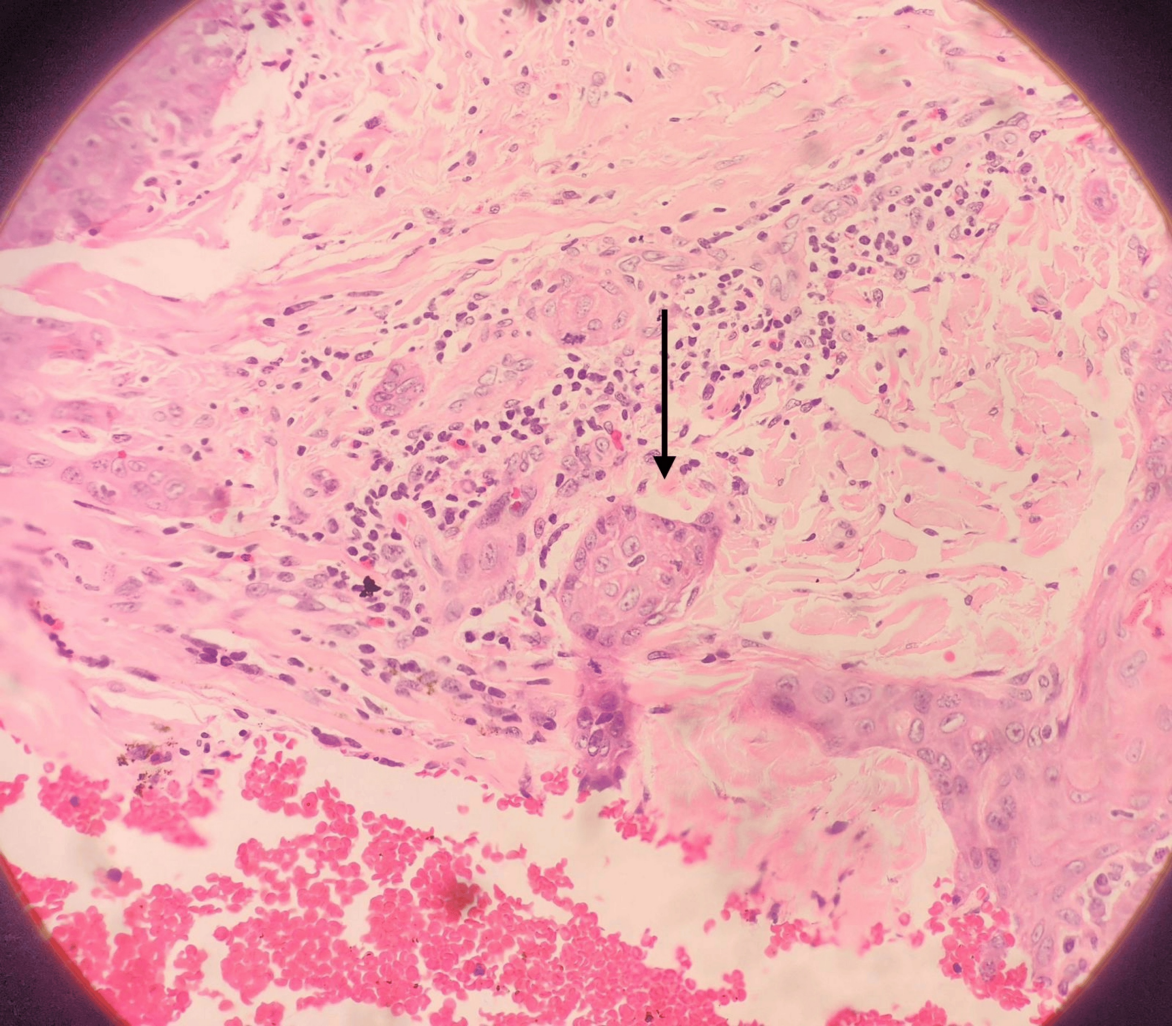
Evidence of keratinisation within tumor islands Arrow indicates evidence of keratinisation within tumor islands, and the surrounding stroma shows moderate inflammatory infiltrate, predominantly lymphocytes.

Histopathological examination revealed epidermal proliferation of neoplastic cellular nests demonstrating keratinization patterns and focal keratin pearl formation, with evidence of tumor cell invasion into the upper dermis (Figure 3). 

## Discussion

This case illustrates the diagnostic difficulties in delineating well-differentiated SCC from HLP and other chronic inflammatory dermatoses.

SCC arising in HLP is rare but documented, with the average time lag between onset of HLP and development of SCC reported as 11 years [5]. A comprehensive review found that malignant transformation occurred across a wide age range, with an average of 61.4 years in women and 51.3 years in men [2]. The chance of neoplastic transformation has been estimated at 0.4-1.2% [6]. In this case, the six-year history of a single lesion with gradual progression and therapeutic refractoriness served as a clinical clue.

Immunohistochemistry markers such as p53, Ki-67, and E-cadherin have demonstrated value in atypical cases for assessing malignant potential. Studies have shown a higher expression of p53 in cases with malignant transformation [4,7-9].

Predisposing factors include chronic inflammation, trauma, and corticosteroid use [10]. In this case, the patient’s prolonged history of corticosteroid use may have contributed to diagnostic delay by masking the malignant changes.

Clinical features that suggest malignant transformation include an increase in lesion size, ulceration, bleeding, pain, and therapeutic refractoriness [2,10]. The differential diagnostic considerations encompassed HLP, chromoblastomycosis, and lupus vulgaris [4].

Surgical excision remains the treatment of choice for cutaneous SCC arising in HLP, with margin assessment being critical due to the risk of local recurrence [6,11]. Adjuvant radiologic staging or sentinel lymph node biopsy may be warranted in high-risk or deeply invasive cases [12].

This case underscores the importance of maintaining a high index of suspicion for malignant transformation in chronic dermatoses, particularly those with atypical clinical features or treatment resistance. Early histopathological evaluation remains crucial for timely diagnosis and appropriate management intervention. Long-term follow-up is vital, not only to monitor for recurrence but also to screen for additional lesions, given the potential for field cancerization in chronic inflammatory dermatoses.

## Conclusions

This case demonstrates the rare but clinically significant occurrence of well-differentiated SCC arising within chronic HLP. The prolonged course of the lesion, its gradual enlargement, and its resistance to conventional therapy were the features that ultimately prompted biopsy and confirmed the diagnosis. It highlights the critical importance of histopathological evaluation in persistent dermatoses with atypical features, as clinical appearance alone may not reliably distinguish benign hypertrophic disease from early malignant change. Prolonged topical corticosteroid use, as seen in this patient, can mask such changes and contribute to diagnostic delay. Regular surveillance of chronic inflammatory skin conditions, with a low threshold for repeat biopsy of non-healing or evolving lesions, is therefore essential for timely detection. Further studies are warranted to establish optimal monitoring protocols and to better define the factors that drive malignant transformation in chronic HLP.
